# Targeted Therapy for HM1.24 (CD317) on Multiple Myeloma Cells

**DOI:** 10.1155/2014/965384

**Published:** 2014-07-17

**Authors:** Takeshi Harada, Shuji Ozaki

**Affiliations:** ^1^Department of Medicine and Bioregulatory Sciences, University of Tokushima Graduate School of Medical Sciences, 3-18-15 Kuramoto, Tokushima 770-8503, Japan; ^2^Department of Hematology, Tokushima Prefectural Central Hospital, 1-10-3 Kuramoto, Tokushima 770-8539, Japan

## Abstract

Multiple myeloma (MM) still remains an incurable disease, at least because of the existence of cell-adhesion mediated drug-resistant MM cells and/or continuous recruitment of presumed MM cancer stem cell-like cells (CSCs). As a new alternative treatment modality, immunological approaches using monoclonal antibodies (mAbs) and/or cytotoxic T lymphocytes (CTLs) are now attracting much attention as a novel strategy attacking MM cells. We have identified that HM1.24 [also known as bone marrow stromal cell antigen 2 (BST2) or CD317] is overexpressed on not only mature MM cells but also MM CSCs. We then have developed a humanized mAb to HM1.24 and defucosylated version of the mAb to adapt to clinical practice. Moreover, we have successfully induced HM1.24-specific CTLs against MM cells. The combination of these innovative therapeutic modalities may likely exert an anti-MM activity by evading the drug resistance mechanism and eliminating presumed CSCs in MM.

## 1. Introduction 

Multiple myeloma (MM) is a plasma cell neoplasm in the bone marrow and is likely to present with hypercalcemia, renal failure, anemia, bone resorption (CRAB), and/or immunodeficiency [1]. Treatment approaches in the management of MM have made a remarkable progress in the recent decades and are comprised of high-dose chemotherapy (melphalan) followed by autologous peripheral blood stem cell transplantation (PBSCT) and novel therapies using proteasome inhibitors and immunomodulatory drugs (IMiDs) [2, 3]. These strategies have improved overall survival of MM patients. However, most patients eventually relapse even after the achievement of complete response [4]. Therefore, other novel therapeutic approaches are strongly needed to further improve the outcome of MM.

Treatment with monoclonal antibody (mAb) has demonstrated the efficacy in several hematological malignancies such as CD20-positive malignant lymphomas and chronic lymphocytic leukemia [5, 6]. The principal mechanisms of its cytotoxic activity are derived from antibody-dependent cell-mediated cytotoxicity (ADCC) and complement-dependent cytotoxicity (CDC) [7]. ADCC is induced when mAb binds to the specific antigen on the surface of malignant cells followed by binding of the Fc domain of the mAb to the Fc receptors on the surface of effector cells. The binding affinity between the Fc domains and the Fc receptors is related to control of fucosylation of N-linked oligosaccharides within the immunoglobulin heavy chain Fc regions [8, 9]. To enhance the binding affinity of mAbs to Fc receptors, defucosylated versions of the mAbs have been developed [9].

In MM, several mAbs with confirmed cytotoxic activity have been developed over the past years [10–12]. The targeted molecules of the mAbs include CS1 [13, 14], CD38 [15], CD138 [16], and CD40 [17]. We have identified a new plasma cell-specific antigen, HM1.24, and developed a humanized anti-HM1.24 mAb (AHM). To enhance the cytotoxic activity of the AHM, we have developed a defucosylated version of the AHM and antibody-drug conjugates (ADC).

In addition, to explore the relevance of cellular immunity against HM1.24, we have investigated the activity of HM1.24 peptide-specific cytotoxic T lymphocytes (CTLs) by using peripheral blood mononuclear cells (PBMCs) and peripheral blood stem cells (PBSC) harvested from MM patients.

In this review, we summarize the targeted therapies for HM1.24 and discuss the perspectives of these new targeted therapies in MM.

## 2. HM1.24 Antigen (CD317)

**Figure 1:**
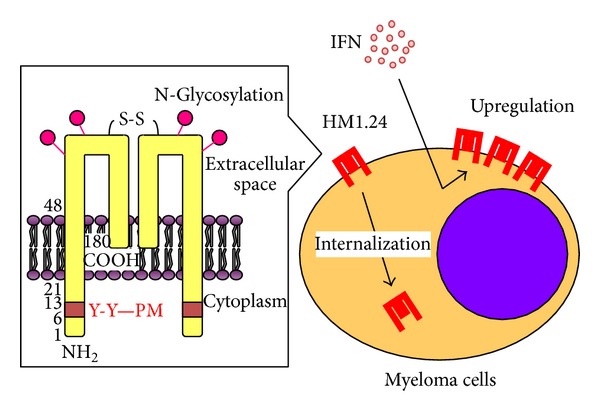
The schema of the structure of HM1.24. HM1.24 is a type II transmembrane glycoprotein that is selectively overexpressed on MM cells as a homodimer with a unique topology. HM1.24 internalizes and localizes to the Golgi apparatus. In the promoter region of HM1.24, there are several* cis*-elements for transcription factors such as IRF-1/2 and ISGF3, and the expression levels of HM1.24 can be upregulated by IFN.

HM1.24 was originally identified as a cell-surface protein that is preferentially overexpressed on MM cells [18]. Later, this protein was found to be identical to bone marrow stromal cell antigen 2 (BST2) and was designated as CD317 [19–22]. This antigen is a type II transmembrane glycoprotein consisting of 180 amino acids with a molecular weight of 29 to 33 kD and is expressed as a homodimer by the disulfide bond (Figure 1). Regarding the topology of HM1.24, the N-terminus is located in the cytoplasm and the transmembrane domain is present near the N-terminus [23]. The cytoplasmic domain contains a Tyr-(X)-Tyr-(X)_3_-Pro-Met sequence motif, which is conserved in mouse, rhesus, and human. The extracellular domain bears two N-linked glycosylation sites, and the C-terminus is modified with a glycosylphosphatidylinositol (GPI) membrane anchor. In addition, HM1.24 is a lipid raft-associated glycoprotein traversing between the cell surface and the Golgi apparatus [23–25].

The HM1.24 gene is located on chromosome 19p13.2 [19]. The promoter region of HM1.24 gene contains the interferon- (IFN-) stimulated response elements such as IFN related factor (IRF)-1/2 and IFN-stimulated gene factor (ISGF) 3, and therefore, the expression of HM1.24 can be upregulated by IFNs especially IFN-*α* [20, 26].

The expression of HM1.24 mRNA is upregulated on both normal and neoplastic plasma cells, and the expression level is increased in symptomatic MM when compared with monoclonal gammopathy of undetermined significance (MGUS) or smoldering MM [27] (http://amazonia.transcriptome.eu/expression.php?geneId=Hs.118110&zone=Hematology-MM). Although the mRNA expression levels vary among primary MM cells [28–30] (http://amazonia.transcriptome.eu/expression.php?geneId=Hs.118110&zone=Hematology-MM), more than 1 × 10^4^ molecules/cell of HM1.24 are detected at the surface of MM cells in more than 85% of patients [31].

During the normal plasma cell differentiation, HM1.24 mRNA is expressed at the highest level in plasmablasts as well as in early plasma cells compared with mature plasma cells [30, 32] (http://amazonia.transcriptome.eu/expression.php?geneId=Hs.118110&zone=PlasmaCell). These findings support the idea that HM1.24 is an intriguing target molecule for immature MM cells or MM cancer stem cells. In fact, we have observed that side population (SP) of MM cells including MM cancer stem cell-like cells (CSCs) expressed HM1.24 at high levels [33].

Several studies have shown that HM1.24 is also expressed on a variety of human tissues and organs such as hepatocytes, pneumocytes, salivary glands, kidney, and vascular endothelium both at the mRNA and protein levels [19, 21, 34]. However, the expression profiles at the protein level in normal tissues have not been clarified yet. In addition, we and other researchers have found that HM1.24 is overexpressed on various cancer cells isolated from breast, lung, kidney, endometrium, and skin [26, 35–43].

The physiological role of HM1.24 remains unclarified; however, recent studies have shown that HM1.24 directly binds to immunoglobulin-like transcript 7 (ILT7) protein and initiates signaling via the ILT-7-Fc*ε*RI*γ* complex [44, 45]. HM1.24 is now termed “tetherin” as a molecule that tethers outgoing virions to the infected cell surface preventing their dissemination [46–48]. However, its biological role in MM cells has not been clarified yet.

## 3. The Development of Anti-HM1.24 mAb Therapy

### 3.1. Mouse Anti-HM1.24 mAb

We first developed a mouse anti-HM1.24 mAb (IgG2a-*κ*) by immunizing Balb/c mice with human MM cells [18]. After fusing spleen cells collected from the immunized mice with myeloma cells and cloning of these fused cells, mAbs that react with the cell surface antigens were obtained after screening the hybridomas by flow cytometry.

To evaluate the specificity of the mouse anti-HM1.24 mAb* in vivo*, we employed a mouse xenograft model using severe combined immunodeficiency (SCID) mice. After establishing subcutaneous tumors of human plasmacytoma(RPMI 8226 cells) in SCID mice, the radiolabeled mouse anti-HM1.24 mAb was injected intravenously, and the biodistribution of the mAb was studied [49, 50]. Our results have shown that the mouse anti-HM1.24 mAb selectively accumulates in the xenograft tumors, suggesting that the anti-HM1.24 mAb has a sufficient specificity for targeting human MM cells* in vivo*.

We next studied the antitumor activity of the mouse anti-HM1.24 mAb. Our* in vitro* experiments have shown that the mouse anti-HM1.24 mAb induces ADCC in the presence of effector cells obtained from mice spleen and CDC in the presence of baby rabbit serum [51]. We next evaluated the* in vivo* efficacy of the mouse anti-HM1.24 mAb using human myeloma xenograft models in SCID mice [51]. The treatment with the mouse anti-HM1.24 mAb has resulted in a decrease of the serum levels of M-proteins and the size of the tumors and has resulted in not only a prolonged survival of the mice but also a cure in some of them.

### 3.2. Humanized Anti-HM1.24 mAb (AHM)

Because the mouse anti-HM1.24 mAb exerted a marked anti-MM activity through the operation of ADCC and CDC machineries, we have established a humanized anti-HM1.24 mAb (AHM, IgG1-*κ*) by grafting the complementary-determining regions [52, 53]. AHM induced ADCC in the presence of human PBMCs against both MM cell lines and MM cells from MM patients, but not CDC in spite of the presence of human serum [31, 52]. The ADCC activity of AHM was increased in a dose-, an effector to target (E/T) ratio-, and HM1.24 expression-dependent fashion. In addition, our* in vivo* experiments have shown that AHM kills MM cells through ADCC [54].

Based on these results, the safety and efficacy of AHM were investigated in a phase I/II clinical study in patients with relapsed or refractory MM in the UK. [55]. Although adverse events were very modest and manageable, the response rate was relatively low in the study. This was considered probably due to the diminished activity of effector cells in this heavily pretreated patient population.

### 3.3. Defucosylated Versions of AHM

In the context of ADCC activity, it has been shown that physiological levels of human serum IgG strongly inhibit the ADCC activity of therapeutic antibodies administered [56]. In addition, a genetic polymorphism of Fc*γ* receptor (Fc*γ*R) IIIa influences the binding affinity between Fc domains of mAb and Fc*γ*RIIIa of effector cells [57–59]. The polymorphism of Fc*γ*RIIIa is present on position 158 [valine (V) or phenylalanine (F)], and patients with homozygous 158 F/F or heterozygous 158 V/F alleles of Fc*γ*RIIIa have been shown to have a lower response rate to rituximab treatment [58, 59]. On the other hand, the binding affinity between the two is controlled by fucosylation in N-linked oligosaccharides within immunoglobulin heavy chain Fc regions [8, 9]. Therefore, defucosylated mAbs might overcome the impaired ADCC activity in terms of a low E/T ratio and a low Fc*γ*RIIIa affinity. To overcome cellular immune deficiency in MM, we have established a defucosylated version of AHM (YB-AHM) with a higher binding ability to Fc*γ*RIIIa [60]. We have found that YB-AHM elicits ADCC more effectively than the parental AHM even with low E/T ratios. Similarly, Tai et al. have shown that Fc-engineered AHM with two amino acid substitutions (S239D/I332E) in the IgG1 Fc portion strongly induces anti-MM activity* in vitro *and* in vivo* [61].

## 4. Augmentation of ADCC Activity by Lenalidomide (Len)

**Figure 2:**
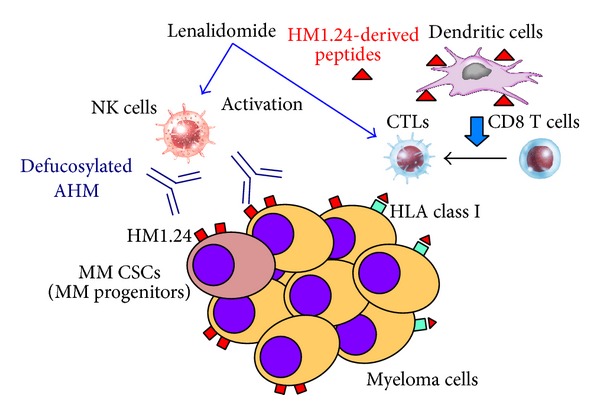
HM1.24-targeted therapy with mAbs and CTLs. Defucosylated AHM induces ADCC activity against MM cells including cancer stem cell-like cells (CSCs) in the presence of human effector cells such as NK cells. On the other hand, functional dendritic cells and HM1.24 peptide-specific CTLs can be induced from PBMCs or PBSC harvests, and these CTLs have the cytotoxic activity against MM cells. Len augments the activity of these cellular immunities.

Len, one of the IMiDs, induces not only direct cytotoxic effects on MM cells but also immunomodulatory, anti-inflammatory, and antiangiogenic effects on the cells surrounding and supporting MM cells in the bone marrow [62]. In particular, Len stimulates the activity of T, NKT, and NK cells and enhances the ADCC activity (Figure 2). For these reasons, Len has been combined with various mAbs including anti-CS1 [63, 64], anti-CD38 [65], and anti-CD20 [66] to enhance the therapeutic efficacy of them.

Tai et al. and our group severally studied the ADCC activity of Fc-engineered AHM or YB-AHM in combination with Len against MM cell lines and MM cells obtained from bone marrow mononuclear cells of MM patients [61, 67]. The results have shown that Len can enhance the ADCC activity of both defucosylated versions of AHM and Fc-engineered AHM.

MM cancer stem cell-like cells (CSCs) have been proposed as responsible for drug resistance and relapse although they are not properly defined yet [68]. Side population (SP) cells have been identified as a drug resistant fraction that contains CSCs in MM [33]. We have found that HM1.24 is highly expressed on the surface of SP cells and that the combination of YB-AHM plus Len effectively reduces the number of SP fractions in MM cell lines [67]. Furthermore, this combination inhibited the clonogenic potential of MM CSCs* in vitro* [67]. Thus, the combination therapy with YB-AHM plus Len might become an effective strategy to target putative MM CSCs (Figure 2).

**Figure 3:**
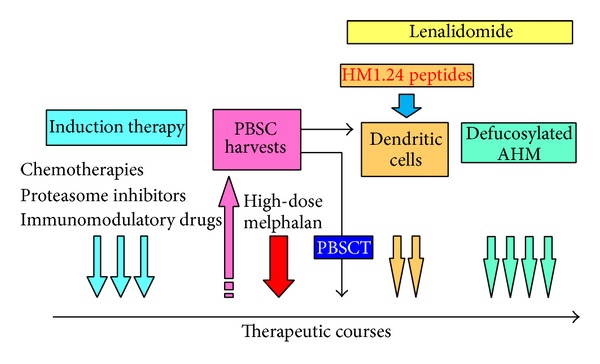
The combination strategy with HM1.24-targeted therapies and the current therapeutic regimen in MM. Induction therapy containing proteasome inhibitors and/or IMiDs and consolidation therapy with high-dose chemotherapy followed by autologous PBSCT induce favorable therapeutic effects; however, the existence of minimal residual disease or MM CSCs is related to relapse and refractoriness. To overcome the drug resistance of MM cells, active immunotherapy with HM1.24-derived peptides and dendritic cells from autologous PBSC harvests and passive immunotherapy with defucosylated AHM might be effective approaches along with lenalidomide in the treatment of MM.

With respect to targeting therapy, the number and function of effector cells are important for eliciting ADCC activity. Therefore, YB-AHM therapy could be a suitable strategy as consolidation and/or maintenance therapy because MM cells have already been reduced in number and the number of effector cells has recovered (relatively high E/T ratio) by this phase (Figure 3).

## 5. HM1.24 mAb-Conjugated ADC

ADC is another approach to enhance the efficacy of mAb therapy. Several ADCs have been developed by the conjugation of mAb with either cytotoxins or radiation emitters to increase the antitumor effect. HM1.24 is a suitable element of ADC because this antigen is internalized from MM cell surface into the Golgi apparatus. Therefore, we have manufactured ADC by using an internalizing mAbs specific to HM1.24 [69]. One of the fully human anti-HM1.24 mAbs, b-76-8, is rapidly internalized after cell surface binding. Thus, ADC consisted of b-76-8 and the analog of the cytotoxic drug maytansine, DM1 [N2′-deacetyl-N2′-(3-mercapto-1-oxopropyl)-maytansine], has been developed. Our results have shown that this ADC significantly elicits the cytotoxic activity against MM cells without effector cells* in vitro* and* in vivo*. Recently, Staudinger et al. have established a novel single-chain immunotoxin, HM1.24-ETA′, by genetic fusion of a HM1.24-specific single chain Fv antibody and a truncated variant of* Pseudomonas aeruginosa* exotoxin A (ETA′) [70].

## 6. HM1.24 Peptide-Specific CTLs

Besides anti-HM1.24 mAb therapy, we have also examined the possibility of HM1.24-specific CTL therapy against MM cells (Figure 2). We selected four HM1.24-derived peptides that possess binding motifs for HLA-A2 or HLA-A24 by using two computer-based algorithms and developed the methods inducing HM1.24 peptide-specific CTLs from PBMCs of healthy donors or PBSC harvests from MM patients in the presence of HM1.24 peptide-pulsed dendritic cells [71]. The experiments* in vitro* have shown that HM1.24 peptide-induced CTLs have the direct cytotoxic activity against MM cells. Several investigators have reported similar results by using HM1.24-derived peptides [72, 73].

Notably, Len has been reported to augment the cytotoxic activity of CTLs in MM [74] including HM1.24-specific CTLs [75]. On the other hand, Herth et al. have recently reported that thalidomide maintenance therapy compromises the HM1.24-specific CTL immunity in MM patients who underwent PBSCT [76]. These results indicate that the cellular immunotherapy targeted for HM1.24 could also be effective in MM, and further studies are warranted to determine whether the IMiDs maintenance therapy with Len or pomalidomide could augment antigen-specific T cell activity.

High-dose chemotherapy followed by autologous PBSCT is considered the most effective consolidation therapy for younger patients with MM. For this procedure, PBSCs are harvested and cryopreserved together with peripheral lymphocytes and monocytes. Therefore, we have investigated the possibility for active CTL therapy by using residual PBSC products after PBSCT. Tarte et al. have previously reported the generation of functional dendritic cells using apheresis products from MM patients [77]. Our results have confirmed that frozen PBSC harvests are useful source for dendritic cells and also for HM1.24-specific CTLs [71]. Thus, we consider that HM1.24-specific cellular immunotherapy could be applied to increase the therapeutic efficacy of autologous PBSCT (Figure 3).

## 7. Conclusion

HM1.24 is an overexpressed antigen on MM cells, and HM1.24-targeted therapies might provide alternative strategies in the management of MM. With regard to mAb therapy, the defucosylated versions of AHM have been established and the synergistic effects have been shown when combined with Len. To further enhance the cytotoxic activity of mAbs, several types of ADC have been developed. Moreover, CTLs specific for HM1.24 have been successfully induced from PBSC harvests obtained from MM patients, and the activity could further be augmented by Len. Most importantly,* in vitro* experiments have shown that some of these approaches are effective for the eradication of MM CSCs.

The treatment paradigm of MM has been dramatically changed since the introduction of autologous PBSCT and novel agents such as thalidomide, lenalidomide, and bortezomib. HM1.24-targeted therapies can be combined with the current therapeutic approaches (Figure 3). Further studies are needed to determine whether these strategies could improve the outcome of MM patients.
